# An unusual case of granulomatous scleromyxedema

**DOI:** 10.1016/j.jdcr.2022.06.022

**Published:** 2022-07-02

**Authors:** Lauren Michelle, Sara Sabeti, Katerina Yale, Brittany Urso, Bonnie Lee, Janellen Smith

**Affiliations:** a School of Medicine, University of California, Irvine, Irvine, California; b Department of Dermatology, University of California Irvine Medical Center, Orange, California

**Keywords:** IVIG, primary cutaneous mucinoses, scleromyxedema, IVIG, intravenous immunoglobulin

## Introduction

Scleromyxedema, also referred to as “generalized and sclerodermoid lichen myxedematosus” or “Arndt-Gottron disease,” is a progressive disease in the spectrum of primary cutaneous mucinoses.^1^ Given its rarity, existing knowledge is primarily derived from case reports and a few retrospective studies. Middle-aged adults are most commonly affected; however, there is no race or sex predominance.^1^ The pathogenesis has not been fully elucidated, but it is hypothesized to be triggered by dysregulated cytokine stimulation of glycosaminoglycan synthesis and fibroblast proliferation.^1^ The 4 main diagnostic criteria include the following: (1) a generalized papular and sclerodermoid eruption; (2) a histologic triad of mucin deposition, fibroblast proliferation, and fibrosis; (3) a monoclonal gammopathy; and (4) the clinical absence of thyroid disease.^2^ Scleromyxedema is commonly associated with an IgG-λ monoclonal gammopathy; however, paraprotein levels do not correlate with disease severity or progression.^1^ Extracutaneous manifestations are common, and previous studies have reported a wide range of systemic involvement, including neurologic, respiratory, gastrointestinal, rheumatologic, ocular, cardiovascular, renal, and hematologic involvement.^3^ Although no standardized treatment guidelines currently exist, first-line therapy involves intravenous immunoglobulin (IVIG) because of its efficacy and tolerability; however, many patients require continued treatment to avoid relapse.^1^ We report a case of this rare disorder with atypical histologic findings.

## Case report

**Fig 1:**
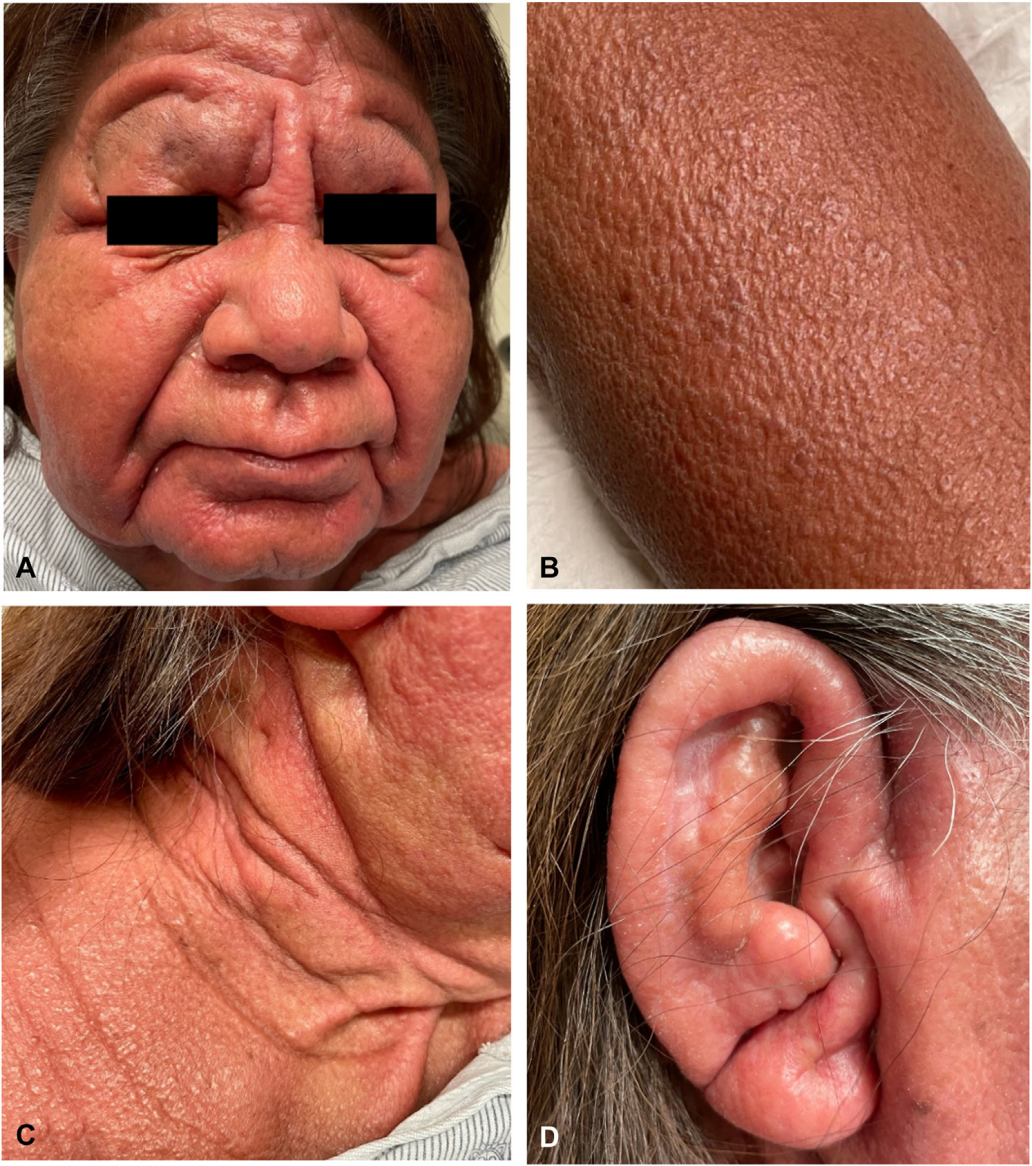
Clinical presentation of scleromyxedema with (**A**) leonine facies, (**B**) waxy, flat-topped papules in linear arrays on the arm, (**C**) exaggerated skinfolds (Shar-Pei sign) on the neck, and (**D**) waxy thickening and narrowing of the external auditory meatus.

**Fig 2:**
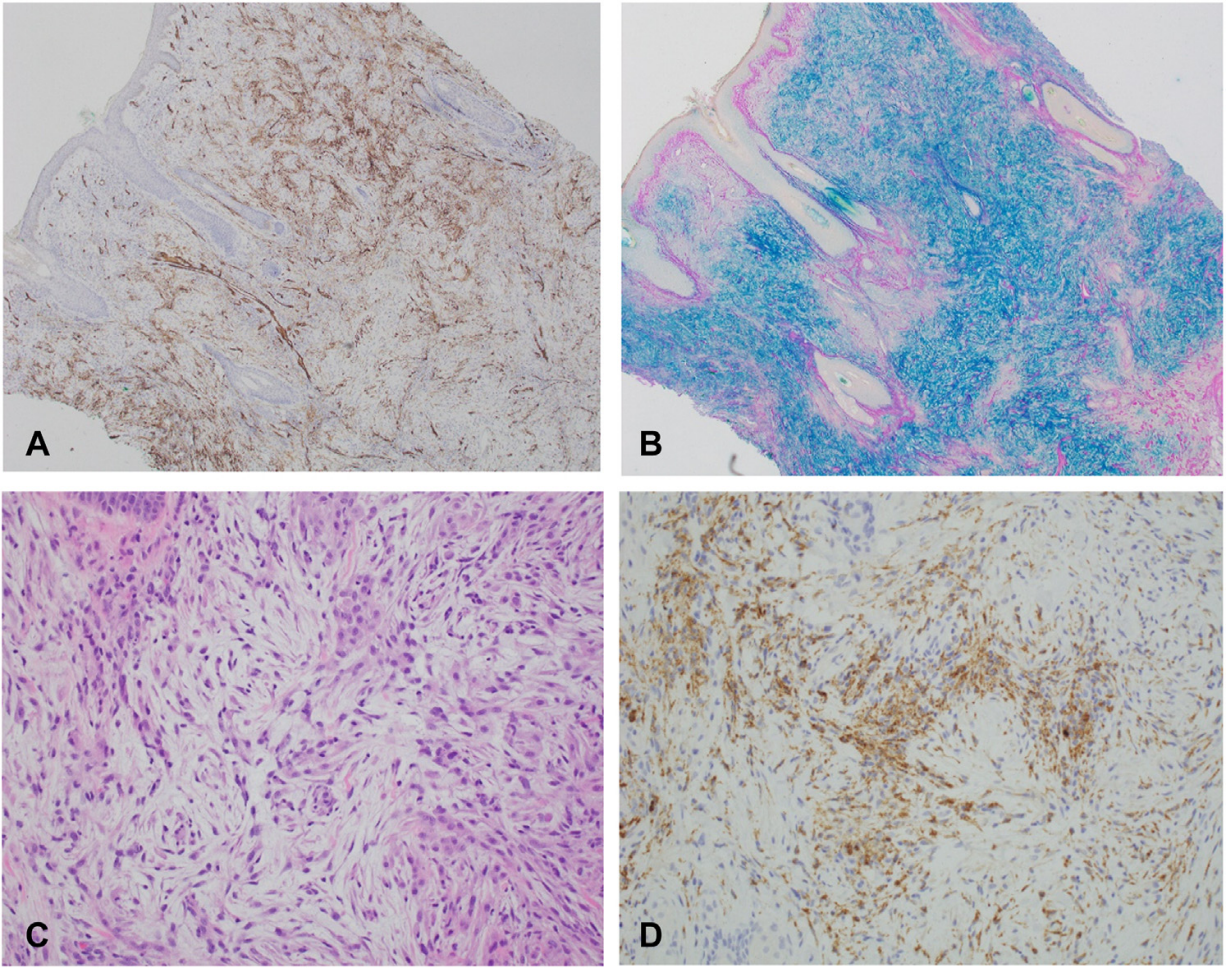
Dermatopathology from a punch biopsy of the right cheek showing (**A**) 4× view of CD34 staining, signifying increased fibroblasts, (**B**) 4× view of colloidal iron staining, showing increased mucin deposition, (**C**) 20× view of hematoxylin and eosin showing granuloma formation, and (**D**) 20× view with CD68 staining highlighting increased histiocytes.(**A**, CD34 stain; original magnification: 4×; **B**, Colloidal iron stain; original magnification: 4×; **C**, Hematoxylin-eosin stain; original magnification: 20×; and **D**, CD68 stain; original magnification: 20×).

**Fig 3:**
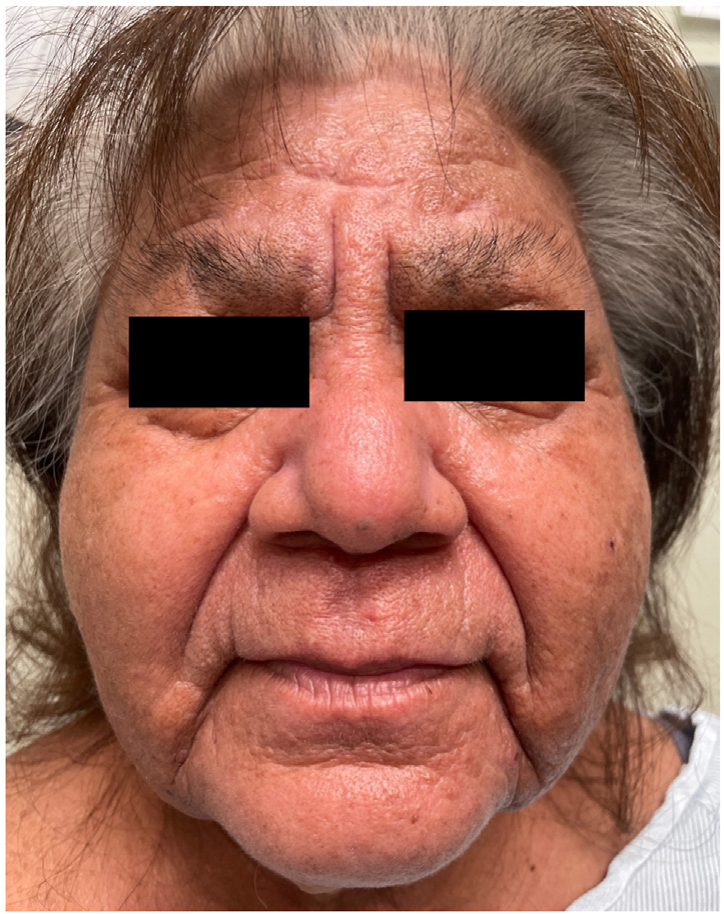
Patient response after 3 months to intravenous immunoglobulin 0.5 gm/kg/d for 4 consecutive days per month.

A 71-year-old Hispanic woman with a history of stable hypothyroidism presented with 12 weeks of progressive, diffuse edema and erythema. Symptoms began with redness, swelling, and pruritus on the face, which rapidly spread to the chest during a Hawaiian vacation, with no precipitating events or medication changes. She was initially trialed on 2 courses of oral prednisone (unknown dose) and high-potency topical steroids, without noticeable improvement, by an outside provider. Review of systems on presentation to our clinic was remarkable for fatigue, dyspnea, diffuse arthralgias, constipation, headaches, and dysphagia. Diffuse waxy red-brown papules coalescing into firm plaques were present on the head, trunk, and extremities, sparing the lower portion of the legs and feet (Fig 1). The patient was also noted to have leonine facies, “Shar-pei sign” on the arms, and “doughnut sign” over the right fifth interphalangeal joint. Laboratories were significant for increased blood urea nitrogen, aldolase, lactate dehydrogenase, and thyroid stimulating hormone, with low free T4. Serum and urine protein electrophoresis showed bands with restricted mobility in the gamma region, which were identified by immunofixation as IgG-κ monoclonal and IgG-λ monoclonal proteins. Kappa and λ free light chains were elevated in the serum, with an increased kappa-lambda ratio. The chest radiograph indicated pulmonary edema, while the echocardiography was normal. No gastrointestinal studies were performed, yet the remainder of the workup for multiorgan involvement was negative. A punch biopsy of the cheek showed a diffuse proliferation of fibroblasts with abundant mucin and interstitial histiocytes highlighted by a CD68 immunostain throughout the dermis, consistent with granulomatous scleromyxedema (Fig 2). Subsequently, the patient was started on IVIG 0.5 gm/kg/d over 4 consecutive days per month with slow improvement in her generalized weakness, dysphagia, and rash over 3 months (Fig 3). Hematology/oncology follow-up revealed no evidence of malignancy.

## Discussion

The diagnosis of scleromyxedema is based on the 4 criteria noted above, initially proposed in 1953 by Montgomery and Underwood,^4^ and revised in 2001 by Rongioletti and Rebora.^2^ The emergence in the literature of atypical cases has challenged these diagnostic standards. In 2017, Nofal et al^5^ proposed updated guidelines to include variations in the clinical, histologic, and ancillary features of scleromyxedema; and more recently, Hazan et al^6^ argued that diagnostic criteria should not exclude the presence of thyroid disease. Our patient’s case is particularly unusual in that she was found to have biopsy-proven scleromyxedema with multiple concurrent atypical features, including concomitant hypothyroidism, biclonal gammopathy, and admixed interstitial granulomatous infiltrate on biopsy.

Several exceptions to the diagnostic criterion of the absence of thyroid disease have been described in the literature. Hazan et al^6^ reported a case of scleromyxedema in a patient with hypothyroidism and suggested that the original classification simply sought to differentiate between mucinosis caused by thyroid abnormalities and that caused by a primary cutaneous mucinous process. Our patient’s thyroid disease was stable on levothyroxine 50 mcg daily. Her clinical symptoms, laboratory findings, biopsy results, as well as her improvement on IVIG all support the diagnosis of scleromyxedema, even in the presence of underlying thyroid dysfunction.

Additionally, our patient’s presentation of scleromyxedema with a biclonal gammopathy and interstitial granulomatous findings is unusual. Only 2 cases of scleromyxedema with biclonal gammopathy have previously been reported.^7^^,^^8^ Shlyankevich et al^7^ described a 54-year-old man who developed a suspected granulomatous drug reaction, which persisted after medication discontinuation, and was found to have scleromyxedema with an interstitial granulomatous pattern with a concurrent biclonal IgG and IgA-λ gammopathy. The patient improved on lenalidomide and dexamethasone. Manousaridis et al^8^ similarly treated a 63-year-old man with refractory scleromyxedema who was found to have biclonal IgG and immunoglobulin M-λ gammopathy. He failed to respond to methylprednisolone alone and saw great improvement on IVIG.^8^ Finally, in a retrospective study of 34 patients, Rongioletti et al^9^ found that an interstitial granuloma annulare-like pattern was present in 23% of the scleromyxedema patients biposied. Although the significance of the granulomatous findings is unknown, it has been proposed that this reaction is an immune response to the monoclonal gammopathy associated with the disease, similar to the granulomatous response to lymphomas, which has been previously suggested.^9^ Current literature does not explain whether the granulomatous histologic variant correlates with a clinical presentation or prognosis but, because of atypical features, may delay diagnosis and treatment initiation. Possible theories for this variant may include disease chronicity, variable triggers, or host defense heterogeneity.

Furthermore, although our patient’s workup was negative for multiorgan involvement, previous reports have shown that extracutaneous involvement can be observed, as mentioned above.^3^ Based on review of systems, further laboratory tests and imaging studies should be performed.

The case presented here further supports the recommendations of previous authors^5^^,^^6^ for updated diagnostic criteria to allow for concurrent thyroid dysfunction and to recognize other variations in clinical or histologic disease presentation. Renewal of diagnostic criteria would assist clinicians in prompt diagnosis and initiating scleromyxedema-directed treatment, thereby reducing morbidity and mortality.

## Conflicts of interest

None disclosed.
